# Mathematical model for the mitigation of the economic effects of the Covid-19 in the Democratic Republic of the Congo

**DOI:** 10.1371/journal.pone.0250775

**Published:** 2021-05-03

**Authors:** Zirhumanana Balike Dieudonné

**Affiliations:** Department of Mathematics and Physics, Institut Supérieur Pédagogique de Bukavu, Bukavu, South-Kivu, Democratic Republic of the Congo; South China University of Technology, CHINA

## Abstract

Since the apparition of the SRAS-Cov-2 in Wuhan in China, several countries have set diverse measures to stop its spread. Measures envisaged include national or local lockdown and travels ban. In the DRC, these measures have seriously prejudiced the economy of the country which is mainly informal. In this paper, a mathematical model for the spread of Covid-19 in Democratic Republic of Congo (DRC) taking into account the vulnerability of congolese economy is proposed. To mitigate the spreading of the virus no national lockdown is proposed, only individuals affected by the virus or suspicious are quarantined. The reproduction number for the Covid-19 is calculated and numerical simulations are performed using Python software. A clear advice for policymakers is deduced from the forecasting of the model.

## 1 Introduction

In December 2019 a novel Coronavirus appeared in Wuhan, China [1]. The international committee for taxonomy of virus has attributed the name SRAS-Cov-2 to that virus [2, 3]. On January 30, 2020; the World Health Organization (WHO) declared it to be an epidemic of international concern [4]. Four months later, the virus was spread out worldwide, only less than 10 countries were not yet touched by the disease. This is why WHO declared it to be a pandemic since March 11, 2020 [4].

Countries affected by this outbreak have envisaged several measures to mitigate its negatives effects on their health care systems. Those measures include national lockdown and cancellation of travels to and from outside their borders [5]. Many low-income countries, including African countries, have also adopted the above measures without taking into account the vulnerability of their economies which rely mainly on the informal system [6].

On 10 March 2020, the first infected individual has been detected in the Democratic Republic of Congo (DRC, [7]). On March 18, 2020; the DRC President has announced draconian measures to mitigate the circulation of the pandemic. These measures include cancellation of flights out and from Kinshasa (the capital city) to other provinces, cessation of school and church activities, suspension of barrooms and restaurant activities, prohibition of gatherings of more than twenty persons, etc. These measures have come into force since March 19, 2020 [8].

However, the aforementioned measures have drastically impacted the national economy. A study in [9] shows that hunger could be deadlier than Covid-19 in low-income countries. This is exactly the case for the DRC. Individuals whose daily income relies on the informal system are not able to strictly respect them. While they were set up to control the epidemic, this economic aspect makes them unappropriated to the Congolese context. In the proposed model, there is no need of shutting down all the country, only suspicious and infected people are quarantined. The meaning of ‘suspicious’ will be clarified in the following section.

Since the apparition of this virus, several models have been proposed to help in comprehension of the spread of this virus. In [10], an improved adaptative neuro-fuzzy inference system using an enhanced flower pollination algorithm by using a salp swarm algorithm was proposed to estimate and forecast confirmed cases of Covid-19 in China. The model was declared effective to predict the number of confirmed cases within ten days. Another model was proposed by [11] to evaluate the effectiveness of the evolution of interventions and self-protection measures, to estimate the risk of partial lifting control measures and to predict the epidemic trend of the virus in mainland China excluding Hubei. The authors concluded that the containment strategies set up by Chinese authorities were effective and advised that relieving personal protection too early may lead to the spread of the disease for a longer time and more people would be infected. Several other methods like the one in [12] for evaluating the risk factors for Covid-19 mortality in China or the one proposed in [13] to describe the multiple transmission pathways in the infection dynamics and emphasize the role of the environmental reservoir and other more models have been developed (see also [14–16]). The model in [12] established that age was the leading risk factor of Covid-19 mortality. People beyond 60 years old were particularly vulnerable to the virus. In [13], the authors found that the environmental reservoir is shaping overall disease risk. The model proposed by [17] uses the early reported data to predict the cumulative number of reported cases to the final size. It implements the major public policies restricting social movement, the identification and the isolation of unreported cases, and the impact of the asymptomatic infectious cases. The authors found that the restrictive measures implemented in Wuhan were salutary if they are implemented at a good moment.

Despite this rich literature on the mathematical modeling of the spread of the SRAS-Cov-2, to my knowledge, there is no model that has been performed to help understanding the spreading of this pandemic in DRC and taking into account the economic impact of the virus on the populations.

To fill in this gap and help to address this challenge, an SEIR model (see [18–20] for details of this model) with additional compartments is proposed in this paper. In this model, full lockdown or national quarantine is not envisaged as it may jeopardize the fragile economic system of the country.

In this work, the following methodology is used: I have added two additional compartments to the well known SEIR model: *Quarantined* and *Hospitalized*. A parameterization of the model is done using the least squares method in Python and some unavailable parameters have been assumed or borrowed from existing literature.

## 2 Description of the model

The population is divided into the following subgroups:

- **Susceptibles (S)**: This compartment comprises individuals who are not yet infected and not immunized against the disease. They are recruited at the rate *θ* and transferred into the Exposed group at the rate *β*.
  In DRC, only one laboratory can declare positive individuals amid a Covid-19 testing. It is the National Institute of Biomedical Research (NIBR) located in Kinshasa, the capital city.
  In many cases, after testing, individuals have to wait for many days before they can get the result as the NIBR is far away from many provinces (for example, Kinshasa is 2000 km from Bukavu) and the transportation system is bankrupted. To prevent potentially infected individuals to spread the disease, authorities should quarantine all suspicious individuals from the susceptible populations at rate *ϵ*. Suspicious individuals are those who have been in contact with an infected person (confirmed by testing) or who have recently sojourned in a highly risky area. Individuals quarantined are therefore not necessarily infected but they present some like Covid-19 symptoms or they have been (or are suspected to have been) in contact with a suspicious or confirmed case.
- **Exposed (E)**: This group contains individuals who are infected but not yet infectious. They move into the Infectious (I) group at a rate *α*.
- **Infectious (I)**: In this group, we have individuals who are infectious, i.e those who are able to spread the disease. In general, such individuals are at the onset of the illness. Thus, they will be transferred into the hospital (H) at a rate *ν* and are removed at rate *η*. This removal is due either to death or recovery without being transferred to the hospital. This will particularly happen for young people who are resilient to the disease and some unbelievers who never accept that the virus is deadly and are currently propagating into the population.
- **Hospitalized (H)**: Two categories of individuals are in this group: those who come from the infectious group (I) and those who come from quarantine (Q). They are respectively recruited at rates *ν* and *σ*. Hospitalized individuals are likewise removed (by recovery or by death) at a rate *μ* amid the virus infection.
- **Quarantined (Q)**: Individuals who are deemed suspicious are placed in quarantine from Susceptible group (S) at rate *ϵ* as mentioned above. Then, individuals are transferred either in hospital at rate *σ* or returned into Susceptible group at the rate *γ* if they are declared negative after testing.
  In this subgroup, no removal due to Covid-19 is not possible because all individuals therein are monitored by the sanitary authorities. Any person in quarantine whose situation becomes complicated is immediately taken to hospital where he or she will die or be cured. The removal rate here is very negligible and I consider it to be zero.
- **Removed (R)**: Removed subjects are either recovered or deceased due to Covid-19.

**Fig 1:**
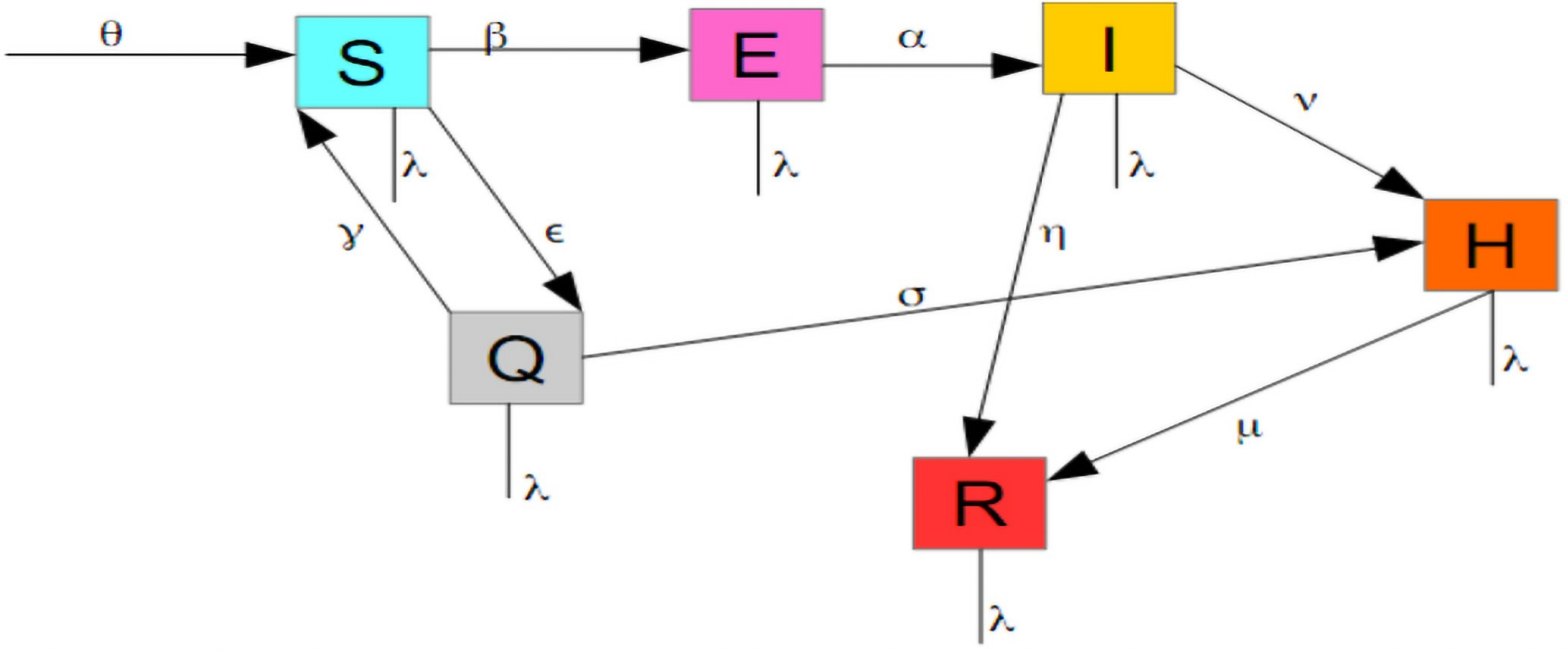
Structure of the proposed model.

In addition, in all compartments, individuals are removed at the same rate λ due to natural death. Fig 1 depicts the structure of this model which yields the following system:

$$\begin{array}{c} \left\{ \begin{array}{cc} \frac{dS}{dt}= & \theta -\beta SI+\gamma Q-(\epsilon +\lambda )S\\ \frac{dE}{dt}= & \beta SI-(\alpha +\lambda )E\\ \frac{dI}{dt}= & \alpha E-(\nu +\eta +\lambda )I\\ \frac{dH}{dt}= & \nu I+\sigma Q-(\mu +\lambda )H\\ \frac{dQ}{dt}= & \epsilon S-(\gamma +\sigma +\lambda )Q\\ \frac{dR}{dt}= & \mu H+\eta I-\lambda R \end{array}\right. \end{array}$$ (1)

### 2.1 Feasible solution and positivity of the solution

In this section, an analysis of the model (Eq 1) will be performed.

It is clear that this system under study satisfies the Lipschitz (see [21]) and theorem 1 (see [22], page 343) conditions. This guarantees the existence and the uniqueness of the solution.

The model is performed for understanding the spread of the SRAS-Cov-2 into a human population. Thus, the parameters are expected to be positive.

Let Ω be the set of all feasible solution to Eq 1. Each solution to a single equation lie in $R_{+}$.

I hence have

$$\begin{array}{c} \Omega =\{\left(S,E,I,H,Q,R\right)\in R_{+}^{6}|N=S+E+I+H+Q+R\} \end{array}$$

where *N* is the total size of the population under study.

In this region, I want

$$\begin{array}{c} \frac{dN}{dt}\geq 0 \end{array}$$ (2)

**Proposition 1**: The set Ω is invariant and attract all solutions in $R_{+}^{6}.$

**Proof**. From the assumption that *N* = *S* + *E* + *I* + *H* + *Q* + *R*, I deduce that

$$\begin{array}{c} \frac{dN}{dt}=\frac{dS}{dt}+\frac{dE}{dt}+\frac{dI}{dt}+\frac{dH}{dt}+\frac{dQ}{dt}+\frac{dR}{dt}. \end{array}$$

Substituting each derivative by the corresponding value from Eq 1 and simplifying, I get:

$$\begin{array}{c} \frac{dN}{dt}=\theta -\lambda [S+E+I+H+Q+R] \end{array}$$ (3)

This equation can be rewritten in the following way:

$$\begin{array}{c} \frac{dN}{dt}+\lambda N=\theta \end{array}$$ (4)

Eq 4 can be solved using an integrating factor.

Multiplying both sides of that equation by e^∫λ*dt*^, I get:

$$\begin{array}{c} {\text{e}}^{\int \lambda dt}[\frac{dN}{dt}+\lambda N]=\theta {\text{e}}^{\int \lambda dt}\Leftrightarrow \frac{d}{dt}[N{\text{e}}^{\int \lambda dt}]=\theta {\text{e}}^{\int \lambda dt}. \end{array}$$

I finally get

$$\begin{array}{c} N=\frac{\theta }{\lambda }+C{\text{e}}^{-\lambda t}. \end{array}$$ (5)

where C is an arbitrary constant. For *t* = 0, we have $C=N_{0}-\frac{\theta }{\lambda }$ which I substitute in Eq 5 to find

$$\begin{array}{c} N=\frac{\theta }{\lambda }+(N_{0}-\frac{\theta }{\lambda })e^{-\lambda t} \end{array}$$

Thus, I have $N\geq \frac{\theta }{\lambda }$ as *t* → + ∞, provided Eq 2.

This establishes that Ω is positively invariant and attracts all solutions in $R_{+}^{6}$.

In addition, it can be proven that the solution to Eq 1 has a positive solution provided the initial data set (*S*_0_, *E*_0_, *I*_0_, *H*_0_, *Q*_0_, *R*_0_)≥0 ∈ Ω.

For example, I have

$$\begin{array}{c} \frac{dS}{dt}=\theta -\beta SI+\gamma Q-\lambda S\geq -\left(\beta I+\epsilon +\lambda \right)S \end{array}$$

That is

$$\begin{array}{c} S\geq {\text{e}}^{-\int (\beta I+\epsilon +\lambda )dt}. \end{array}$$

The right side of this inequality is always positive, that is the function S is also positive and consequently its initial value.

A similar computation can show that

$$\begin{array}{c} E\geq K_{1}{\text{e}}^{-(\alpha +\lambda )t},\;\;I\geq K_{2}{\text{e}}^{-(\nu +\eta +\lambda )t},\;\;H\geq K_{3}{\text{e}}^{-(\mu +\lambda )t},\;\;Q\geq K_{4}{\text{e}}^{-(\gamma +\sigma +\lambda )t},\;\;R\geq K_{5}{\text{e}}^{-\lambda t} \end{array}$$

where *K*_*i*_, (*i* ∈ {1, ⋯, 5}) are arbitrary constants.

### 2.2 The disease free equilibrium (DFE)

From Eq 1, I set *f*_*i*_ = 0, (*i* = 1, ⋯, 6) to get the new algebraic system below.

$$\begin{array}{c} \left\{ \begin{array}{ccc} \theta -\beta SI+\gamma Q-(\epsilon +\lambda )S & =0 & \left(\text{a}\right)\\ \beta SI-(\alpha +\lambda )E & =0 & \left(\text{b}\right)\\ \alpha E-(\nu +\eta +\lambda )I & =0 & \left(\text{c}\right)\\ \nu I+\sigma Q-(\mu +\lambda )H & =0 & \left(\text{d}\right)\\ \epsilon S-(\gamma +\sigma +\lambda )Q & =0 & \left(\text{e}\right)\\ \mu H+\eta I-\lambda R & =0 & \left(\text{f}\right) \end{array}\right. \end{array}$$ (6)

From equations (a) to (f), I successively get

$$\begin{array}{l} S^{*}=\frac{\theta }{\epsilon +\lambda },\;E^{*}=\frac{\beta SI^{*}}{\alpha +\lambda },\;I^{*}=\frac{\alpha E^{*}}{\nu +\eta +\lambda },\\ \begin{array}{c} \begin{array}{c} H^{*}=\frac{\nu I^{*}+\sigma Q^{*}}{\mu +\lambda },\;Q^{*}=\frac{\epsilon S^{*}}{\gamma +\sigma +\lambda }\;\text{and}\;R^{*}=\frac{\mu H^{*}+\eta I^{*}}{\lambda } \end{array} \end{array} \end{array}$$ (7)

If *E* = 0 (that is *β* = 0) and *ϵ* = 0, then *I* = *H* = *Q* = *R* = 0 and accordingly, the DFE is $P_{0}(\frac{\theta }{\lambda },0,0,0,0,0).$ In this case, there is no disease as no infectious individuals are within the population.

If *ϵ* ≠ 0, the situation is such that suspicious are quarantined but they are neither infectious nor confirmed infected. This is consistent with the logic of the model: individuals with like Covid-19 symptoms are quarantined waiting for the testing results from the NIBR. The corresponding DFE is $P_{0}(\frac{\theta }{\lambda +\epsilon },0,0,0,\frac{\epsilon \theta }{\lambda (\lambda +\epsilon )},0)$ as λ never vanishes.

### 2.3 The basic reproduction number

The infection components in this model are E, I, H and Q. In accordance with the notations in [13, 16, 23], the new infection matrix *F* and the transition matrix *V* are given by

$$\begin{array}{c} F=(\begin{array}{cccc} 0 & \frac{\beta \theta }{\lambda +\epsilon } & 0 & 0\\ 0 & 0 & 0 & 0\\ 0 & 0 & 0 & 0\\ 0 & 0 & 0 & 0 \end{array})\;\text{and}\;V=(\begin{array}{cccc} \alpha +\lambda & 0 & 0 & 0\\ -\alpha & \nu +\eta +\lambda & 0 & 0\\ 0 & -\nu & \mu +\lambda & -\sigma \\ 0 & 0 & 0 & \sigma +\gamma +\lambda \end{array}) \end{array}$$

The basic reproduction number of the virus modeled by Eq 1 is then defined as the spectral radius of the next generation matrix *FV*^−1^, i.e

$$\begin{array}{c} R_{0}=\frac{\alpha \beta \theta }{\left(\epsilon +\lambda )(\alpha +\lambda )(\nu +\lambda +\eta \right)} \end{array}$$ (8)

If the inequality $R_{0}\leq 1$ is satisfied, then no epidemic outbreak is possible, otherwise an epidemic occurs.

If *ϵ* = 0, then the fifth equation of Eq 1 is removed from the infection components (that is, the model comprises only three infection components: E, I and H).

Summing equations (b) and (c) of the Eq 6 I get the following:

$$\begin{array}{c} \beta S^{*}I^{*}-\left(\alpha +\lambda \right)E^{*}+\alpha E^{*}-\left(\nu +\eta +\lambda \right)I^{*}=0 \end{array}$$

After substituting I* from Eq 7 and simplifying, we get:

$$\begin{array}{c} \frac{\beta \alpha S^{*}}{\nu +\eta +\lambda }-\left(\alpha +\lambda \right)=0 \end{array}$$

as E* ≠ 0.

This finally yields

$$\begin{array}{c} S^{*}=\frac{\left(\alpha +\lambda )(\nu +\eta +\lambda \right)}{\beta \alpha }. \end{array}$$ (9)

Provided Eq 2 and its result, I have

$$\begin{array}{c} S^{*}\geq \frac{\theta }{\lambda } \end{array}$$

that is

$$\begin{array}{c} \frac{\left(\alpha +\lambda )(\nu +\lambda +\eta \right)}{\alpha \beta }\geq \frac{\theta }{\lambda }. \end{array}$$

Hence, I get

$$\begin{array}{c} 1\geq \frac{\alpha \beta \theta }{\lambda (\alpha +\lambda )(\nu +\lambda +\eta )}. \end{array}$$

I set

$$\begin{array}{c} R_{0}=\frac{\alpha \beta \theta }{\lambda (\alpha +\lambda )(\nu +\lambda +\eta )} \end{array}$$ (10)

the new reproduction number.

This is exactly the spectral radius of the new next generation matrix obtained after ignoring the fifth equation of Eq 1 in the infection compartments.

Thus, *ϵ* stands for a control on the spread of the disease within the susceptible population.

### 2.4 Local stability

I examine the local stability of the DFE by the mean of the jacobian matrix of the functions *f*_*i*_, *i* = 1, ⋯, 6 (where *f*_*i*_ are the functions in the right side of Eq 1) at that point. I therefore have the jacobian below

$$\begin{array}{c} J_{f}=(\begin{array}{cccccc} -J_{1} & 0 & -\beta S & 0 & \gamma & 0\\ \beta I & -(\alpha +\lambda ) & \beta S & 0 & 0 & 0\\ 0 & \alpha & -J_{2} & 0 & 0 & 0\\ 0 & 0 & \nu & -\mu +\lambda & \sigma & 0\\ \epsilon & 0 & 0 & 0 & -J_{3} & 0\\ 0 & 0 & \eta & \mu & 0 & -\lambda \end{array}) \end{array}$$

where *J*_1_ = βI* + λ + *ϵ*, *J*_2_ = *ν* + *η* + λ, *J*_3_ = *σ* + *γ* + λ.

This jacobian evaluated at the DFE yields

$$\begin{array}{c} J_{f}\left(P_{0}\right)=(\begin{array}{cccccc} -(\lambda +\epsilon ) & 0 & -\beta \frac{\theta }{\lambda +\epsilon } & 0 & 0 & 0\\ 0 & -\lambda & \beta \frac{\theta }{\lambda +\epsilon } & 0 & 0 & 0\\ 0 & 0 & -\lambda & 0 & 0 & 0\\ 0 & 0 & 0 & -\lambda & 0 & 0\\ \epsilon & 0 & 0 & 0 & -\lambda & 0\\ 0 & 0 & 0 & 0 & 0 & -\lambda \end{array}) \end{array}$$

has all its eigenvalues negative whether *ϵ* is null or not.

Thereby, the DFE is locally asymptotically stable.

## 3 Results and discussion

**Fig 2:**
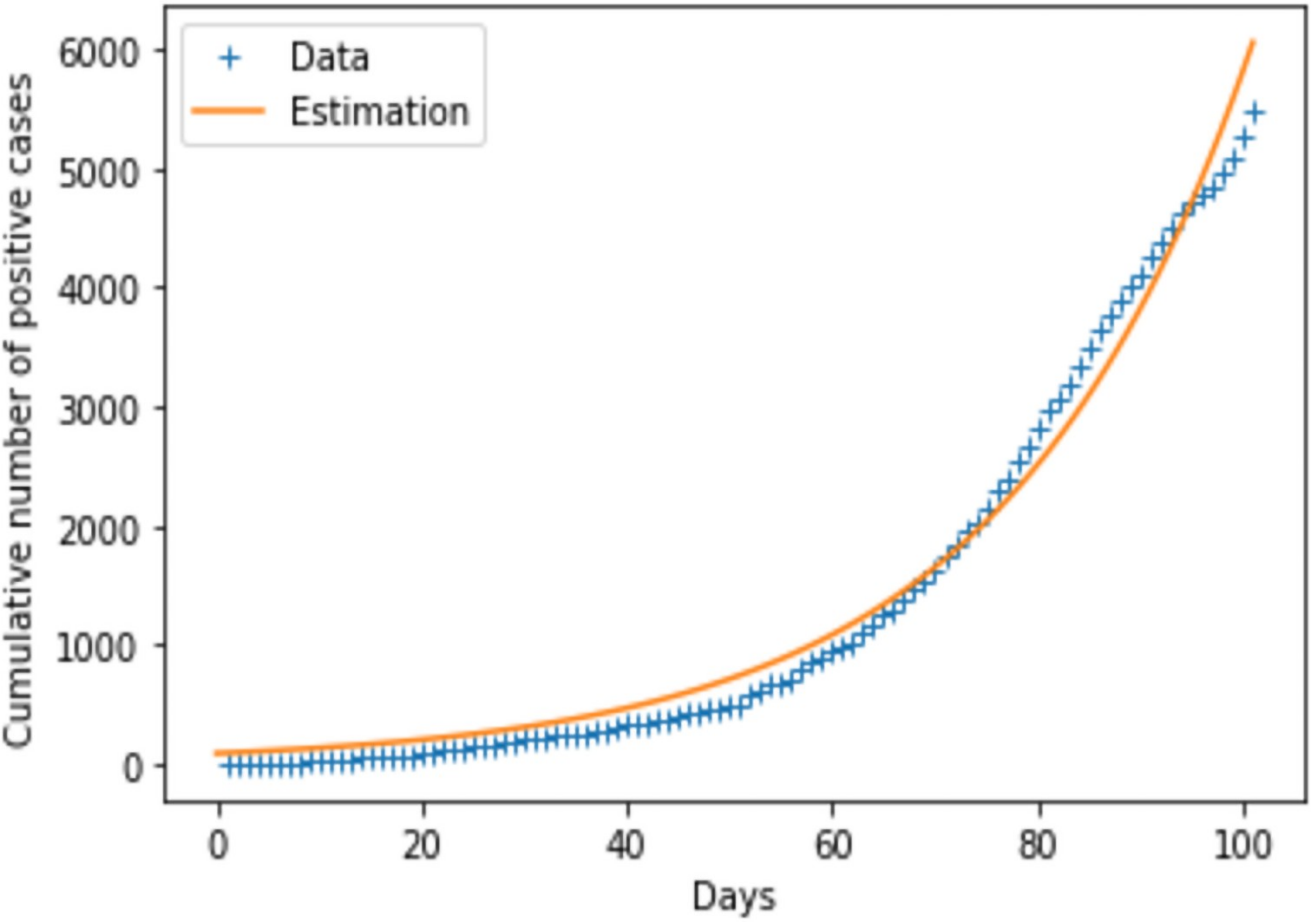
Fitting data to the model for parameters estimation.

This section is devoted to numerical simulations and discussion of the results. One hindrance here is the lack of data related to the Covid-19 in DRC. For example, data on the number of tests performed on a daily basis are not available. The NIBR doesn’t make them available even when they are requested. To evade this obstacle, I used data available on online [24] and made an estimate for some other parameters. The Fig 2 depicts how the model fits the data collected for the 100 first days of Covid-19 crisis in DRC online [24]. Due to lack of available data in DRC, some parameters have been reasonably assumed (without either overlooking or exaggerating them). Provided some parameters after fitting the model with data, some other parameters have been calculated and others were borrowed to [13]. To determine the value of the calculated parameters, I proceed in the following way: At the beginning of the epidemic, *E* = *I* and second equation of Eq 1 becomes

$$\begin{array}{c} \frac{dI}{dt}=\left(\alpha -\nu -\eta -\lambda \right)I \end{array}$$ (11)

whose solution is

$$\begin{array}{c} I=k{\text{e}}^{-(\nu +\eta +\lambda -\alpha )t} \end{array}$$ (12)

where *k* is an arbitrary constant. Using the value got from data fitting, I get

$$\begin{array}{c} \nu +\eta +\lambda -\alpha =4.1987\times 10^{-2}. \end{array}$$

Since the value of λ is known from the data on life expectancy in DRC ($\lambda =\frac{1}{12\times \text{Life}\;\text{expectqncy}}$, see [18, 25] for details) and provided there are some others parameters borrowed from [13], I get the values in Table 1.

**Table 1:** Parameters used in the model.

Parameters	Description	Values	Sources
*θ*	Population influx rate	271.4 day^-1^	[13]
*β*	Transmission rate	5445 × 10^−6^ pers^-1^ day^-1^	Calculated
$\frac{1}{\alpha }$	Incubation period	14 days	[13]
λ	Natural death	0.001378 pers day^-1^	Calculated
*ν*	Hospitalization rate	0.0097 person day^-1^	Assumed
*η*	Removal (recovery and death) rate	0.01428 pers day^-1^	Data
*σ*	Transfer rate from Q to H	$\frac{1}{14}$ day^-1^	Assumed
*ϵ*	Quarantine rate	Varying (pers day^-1^)	This study
*γ*	Transfer rate from Q to S	$\frac{1}{14}$ day^-1^	Assumed
*μ*	Removal rate from hospital	$\frac{1}{28}$ day^-1^	Assumed

Using the values of parameters like provided in Table 1, a simulation of the model has been performed in Python. The model is designed such that Susceptible stratum collapses as early as possible. This makes the Exposed and Infectious groups to explode quickly.

**Fig 3:**
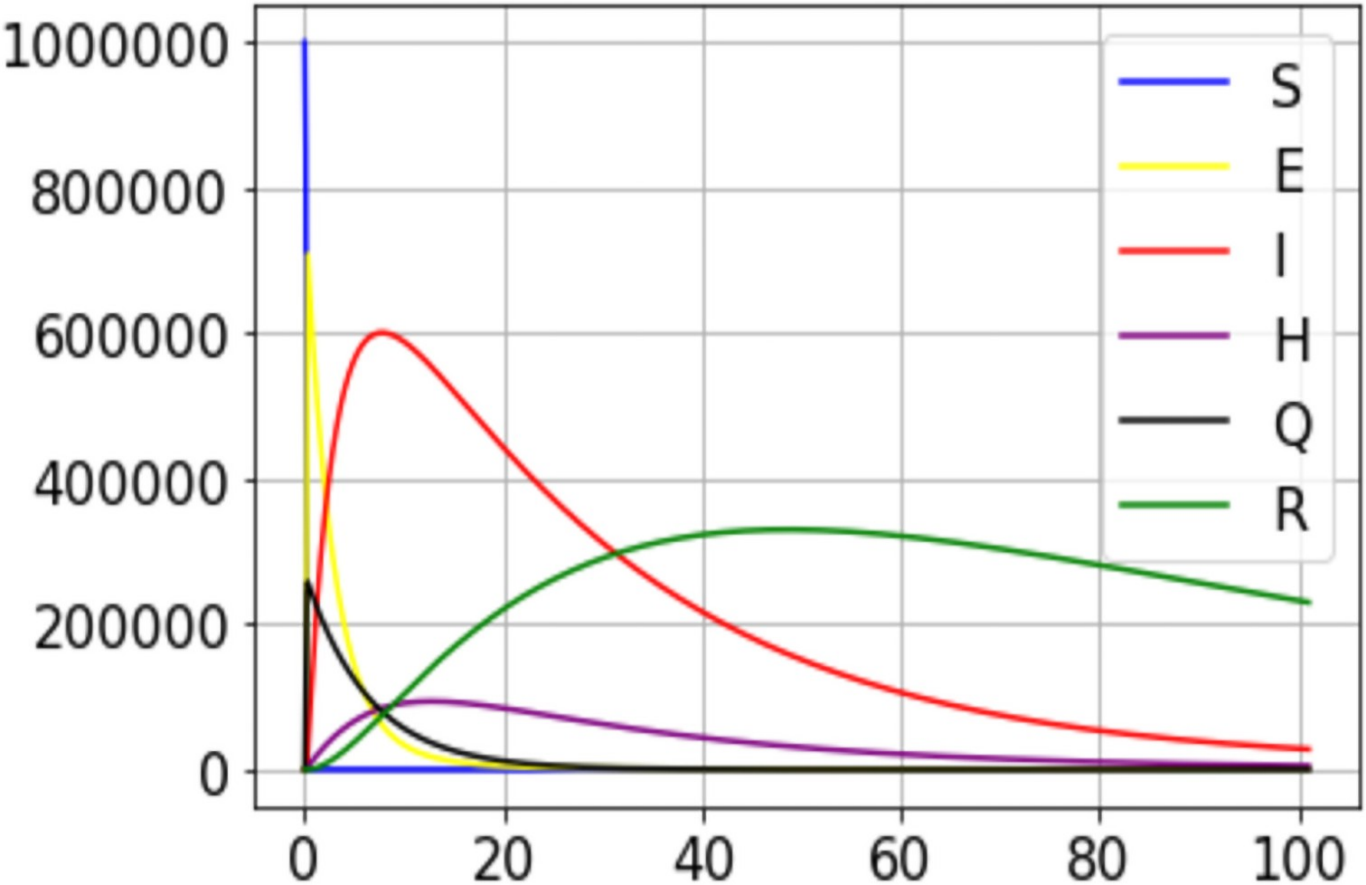
A simulation result for the outbreak using data from the 100 first days data from DRC (*ϵ* = 1.5).

**Fig 4:**
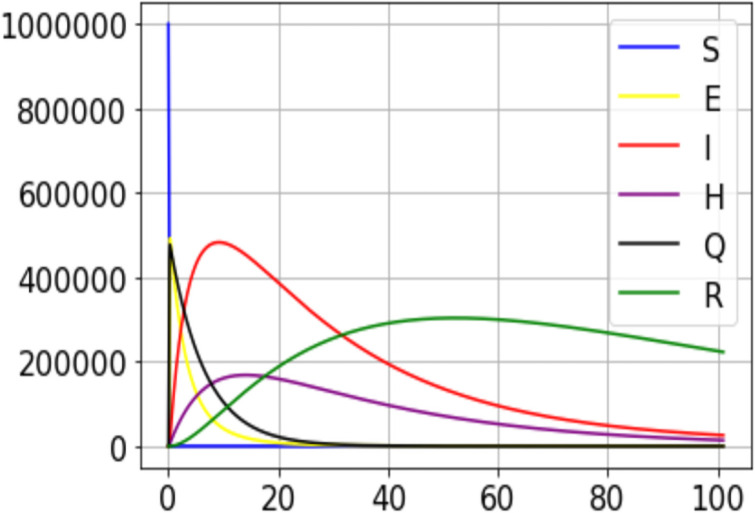
A simulation result for the outbreak using data from the 100 first days data from DRC(*ϵ* = 3.1).

The impact of *ϵ* can be evaluated on Figs 3 and 4. When the number of individuals in group Q is high, the number of positive cases drops but the number of individuals hospitalized increases. This is proof that organized quarantine allows rapid detection of positive cases and enables the timely management of patients who need to be hospitalized.

The policy of the government is the national lockdown to stop the spread of the Covid-19 within populations. However, such measure is more likely to increase severe poverty in the country as the major part of the economy is informal.

This model suggests to only quarantine people with like Covid-19 symptoms or those who are affected by the virus.

Assume that all the population is quarantined according to the current policy of the government. Then, in the model we get *S* = *Q*. Of course, this diminishes the contagion rate yet, it also means that no economic activity is done. As result, 70% of Congolese households (according to [6]) relying on a daily revenue will not be able to leave their home for work. It is therefore clear that this measure will affect those families whose subsistence comes from daily income and jeopardize all the national economy.

However, if the idea developed in this model is applied, only three subgroups of individuals will be prohibited to leave their places: Infectious, Quarantined (as I have defined this concept in the description of the model), and Hospitalized. Looking at Figs 3 and 4, none of these groups has its peak at the maximum of the sample studied. In other words, it will not be possible to see the entire population concentrated in these three groups exclusively. Hence, it means that one part of the population will be active (the largest part, by the way) and the other part in one (made up of the three critical groups) will be deprived of any activity. Economically, the non-isolated part of the population will be able to continue their activities.

## 4 Conclusion

The mathematical model proposed in this paper has been designed by taking into account the vulnerability of the economy of the Democratic Republic of the Congo and other low-income countries. Since the country does not have enough means to control the epidemic from Kinshasa as is the case to date, I propose that the management of this crisis be decentralized. Thus, each provincial government will be able to set up a team responsible for tracking down suspect people and quarantine them while waiting for the results of the NIBR tests. That procedure makes it quick to get the result and people who were mistakenly quarantined (because they were deemed suspicious) will quickly be relaxed to pursue their activities. To achieve this, the government should accelerate the testing operations for quarantined people.

Since the national economy is informal, this measure will help to fight the virus in keeping the economy in motion.

In addition to these containment strategies, the government should ensure that people allowed to move wear correctly the masks and respect social distancing.
